# Gait Medicine: an interdisciplinary field centered on walking as a vital sign

**DOI:** 10.3389/fpubh.2026.1895889

**Published:** 2026-09-07

**Authors:** Katsuya Hisamichi, Kazuhito Nagasaki, Masuomi Tomita, Naoto Takeda, Naoki Tanaka, Shohei Mukai, Kyota Kikuchi

**Affiliations:** 1 Shimokitazawa Hospital, Setagaya, Japan; 2 Department of Vascular Surgery, Shimokitazawa Hospital, Setagaya, Japan; 3 Diabetic Center, Shimokitazawa Hospital, Setagaya, Japan; 4 Department of Rehabilitation, Shimokitazawa Hospital, Setagaya, Japan; 5 Shimokitazawa Hospital, Gait Analysis Laboratory, Setagaya, Japan; 6 Footwear Division, SOL&Co. Ltd, Shibuya, Japan; 7 Podiatry Center, Shimokitazawa Hospital, Setagaya, Japan

**Keywords:** autonomous mobility, Gait Medicine, gait speed, healthy aging, interdisciplinary, three-layer model, walking

## Abstract

Walking speed predicts survival, cognitive decline, and disability in older adults with a precision comparable to that of blood pressure—yet no single discipline organizes itself primarily around walking. The result is a clinical vacuum: when walking deteriorates from the convergence of weak feet, eroding confidence, and an unwalkable neighborhood, patients have no specialist to consult and clinicians have no integrative framework to apply. We propose Gait Medicine as an interdisciplinary field organized around a three-layer model—somatic (foot structure, neuromuscular function, sensory-perceptual systems, cognition, affect, and motivation), environmental (footwear and built environment), and meaning (social participation, cultural significance, and evolutionary origin)—with autonomous mobility as its superordinate concept, explicitly encompassing those who cannot walk. We define the field’s conceptual boundaries, distinguish it from five adjacent disciplines, respond to three anticipated objections, and outline clinical implications including a Gait Clinic prototype and urban policy applications. The three-layer coupling model generates falsifiable predictions about integrative interventions that can be tested empirically. Gait Medicine does not claim to have discovered walking. It argues that medicine has not yet organized a discipline around it—and that the evidence, the patients, and the technology now make doing so both feasible and necessary.

## 1 Introduction

In 2011, Studenski et al. pooled individual-level data from nine cohorts and found that gait speed predicts survival in older adults with a precision comparable to that of blood pressure or ejection fraction (1). The result did not emerge in isolation. Nearly a decade before, Verghese et al. had demonstrated that gait abnormalities predict non-Alzheimer dementia (2), and Montero-Odasso et al. confirmed gait velocity as a single predictor of adverse events in otherwise healthy seniors (3). The cumulative evidence is hard to dismiss. Walking is a vital sign.

And yet no single discipline organizes itself primarily around walking.

This is not for lack of attention. Rehabilitation medicine has developed sophisticated, neuroplasticity-informed approaches to gait recovery after stroke and other neurological injury (4). Neurology treats gait disorders as a structured diagnostic domain in their own right, with established frameworks for distinguishing sensory, parkinsonian, and frontal gait syndromes (5). Geriatric medicine has produced comprehensive clinical guides to gait disturbance in older adults, integrating neurological, musculoskeletal, and orthostatic contributions (6). Each of these fields treats gait extensively and, within its own scope, rigorously. Nor is this attention confined to clinical medicine: biomechanics, motor control, and the exercise sciences have produced the greater part of what is known about how walking is generated and how it changes across the lifespan.

What none of them does is organize itself around walking as the central concern. Consider the situation from the clinician’s perspective. The foot belongs to podiatry. The gait cycle, when disrupted by stroke or Parkinsonism, is addressed within rehabilitation medicine and neurology. Balance falls to neurology or otolaryngology, depending on the suspected etiology. Fear of falling—a distinct psychological construct, not merely a symptom of past falls—is the province of geriatric psychiatry (7, 8). The sidewalk itself, perhaps the most fundamental piece of walking infrastructure, belongs to urban planning. Each discipline addresses walking competently within its own frame. None makes walking the organizing axis around which the others are coordinated.

The consequence is something practicing clinicians will recognize immediately. A 78-year-old woman presents saying she has been walking less. Not because of a stroke. Not because of a hip fracture. But because her forefoot hurts, her antihypertensive makes her dizzy in the mornings, she fell once last winter and has been anxious about it ever since, and the intersection near her apartment was redesigned in a way that leaves too little time to cross. Which specialist should she see? In most health systems, the honest answer is: nobody in particular.

This fragmentation is not merely inconvenient. It is a structural failure in the way medicine is organized. Walking is the most common form of physical activity across all human populations. It is the primary means by which people access social life, economic activity, and community belonging (9, 10). It is an evolutionarily acquired trait that is among the defining characteristics of our species, with bipedal locomotion conferring substantial energetic advantages over quadrupedal movement (11). A medical system that acknowledges all of this and still assigns walking to no organizing axis has a blind spot where a vital sign should be.

The timing is not arbitrary. Wearable accelerometers and smartphone-based gait monitoring have only recently matured to the point where continuous, ecologically valid gait measurement is feasible outside laboratories. At the same time, the population aged 65 and over is on track to double by 2050—and with it, the clinical cost of leaving walking to no organizing axis. The COVID-19 pandemic sharpened the point, producing in a few months the kind of enforced mobility restriction that revealed how much of late-life function rests on the simple act of going outside. None of these by itself would warrant a new field. Together, they make one harder to defer.

We propose that the blind spot has a name. The name is Gait Medicine.

## 2 Defining Gait Medicine

Before defining the field itself, we clarify four terms that recur throughout this paper and that are frequently used interchangeably in the broader literature: movement, mobility, walking, and gait. Left undifferentiated, these terms obscure rather than clarify the field’s scope.

Movement is the broadest construct: any change in the spatial configuration of the body or its parts, encompassing everything from a hand gesture to a marathon stride. Mobility is narrower. It denotes the capacity to move through, and interact with, one’s environment in order to accomplish a purpose—reaching a destination, retrieving an object, changing position within a room. Mobility is defined by function and intent, not by the means through which that function is achieved. Walking, or ambulation, is one strategy for accomplishing mobility: the specific pattern of bipedal locomotion by which most, but not all, humans move through the world. Gait is narrower still. It refers to the biomechanical and neurophysiological organization that underlies walking—the coordinated sequence of joint angles, muscle activations, and neural timing that produces a walking step.

These four terms therefore stand in a hierarchical relationship: gait is the mechanical substrate of walking; walking is one strategy for achieving mobility; mobility is one domain of movement, specifically the domain oriented toward purposeful interaction with the environment. This hierarchy clarifies why we name this field Gait Medicine while defining its scope in terms of mobility rather than gait alone. The field is anchored empirically in gait—the level at which the evidence reviewed in Section 1 is most extensive, and the level at which clinical assessment tools are most developed—while its conceptual scope extends to the broader construct of mobility, of which gait is the most common but not the only expression. A person who propels a manual wheelchair, or who navigates using a powered mobility device, is engaged in mobility in the full sense defined above: purposeful, self-directed movement through the environment. They are not engaged in gait, because gait specifically denotes the bipedal locomotor pattern. The name Gait Medicine reflects the field’s empirical anchor and its point of clinical entry; it does not restrict the field’s conceptual boundary, which this section develops below.

With this hierarchy established, we can now define the field itself. Gait Medicine is an interdisciplinary medical field that regards walking as a fundamental physiological function, a foundation of mental health, a means of social participation, a basis of urban life, a cultural practice, and an evolutionarily acquired species-defining trait. It aims to extend healthy life expectancy and secure personal autonomy through the maintenance, enhancement, and restoration of walking ability.

Two clarifications are necessary at the outset.

First, Gait Medicine is not gait analysis. Gait analysis is a measurement technique. Gait Medicine is a field—a way of organizing clinical thinking, research, and policy around walking as the central concern. The distinction is the same as the one between electrocardiography and cardiology.

Second, Gait Medicine does not equate health with the ability to walk. It positions autonomous mobility—the capacity to move through the world on one’s own terms, by whatever means—as the superordinate concept. Walking is autonomous mobility’s most universal form, not its only form. Where walking cannot be restored, the field encompasses the optimization of alternative modes of movement. This is not a hedge inserted for appearances. It is a conceptual requirement. Any field claiming to address human mobility while excluding wheelchair users and amputees would be neither comprehensive nor honest.

One implementation question deserves a direct answer. Gait Medicine as defined here does not require a new medical specialty with its own licensing and board certification. That kind of institutional creation takes decades, and the political obstacles alone would exhaust the case before it began.

What we propose instead is a framework that existing specialists apply within their current scope. The podiatrist fitting an orthotic now asks how that intervention couples to the patient’s neighborhood and social world. The geriatrician treating fear of falling brings in someone who understands the patient’s crossing—not because interdisciplinary care is fashionable, but because the patient’s problem straddles disciplines whether we acknowledge it or not.

The institutional form follows from the principle, not the other way around. A Gait Clinic is one expression of it. So is a research consortium, a policy framework, or a clinical decision tool embedded in an electronic health record. What matters is the organizing axis.

### 2.1 Methodological approach

This paper develops Gait Medicine through theory synthesis—a mode of conceptual work in which concepts and findings drawn from multiple, previously disconnected literatures are integrated into a novel organizing framework. Theory synthesis is a recognized strategy of theory development, cataloged among the established approaches to constructing nursing and health science theory (12) and articulated as one of the principal templates for conceptual articles, in which the contribution arises not from new empirical data but from a reorganization of existing knowledge under a unifying logic (13). As that literature emphasizes, a conceptual article must rest on an explicit research design, and the selection of theories and their role in the analysis must be stated and justified rather than left implicit. We make that design explicit here.

The literature underpinning this synthesis was assembled from a standing body of work accumulated across the authors’ clinical and research practice, supplemented by targeted searches of PubMed and Google Scholar where specific claims required current evidence, and by sources recommended by co-authors from their respective specialties. We did not employ a predefined search string or date range, and we do not present this as a systematic or scoping review; the aim was not an exhaustive census of a defined field but the identification of works that anchor the concept of walking as a vital sign, represent the adjacent disciplines under comparison, and connect to the clinical observations the framework seeks to organize. Sources were selected collaboratively across the author group rather than by a single author.

The five disciplines against which Gait Medicine is delineated in Section 4—rehabilitation medicine, sports medicine, podiatry, public health, and geriatrics—were chosen as those most frequently encountered, in the authors’ clinical experience, at the point where a deteriorating-walking presentation crosses disciplinary boundaries. Other candidate fields, including neurology, orthopedics, and biomechanics, were considered. Neurology and orthopedics were not treated as absent from the discussion but as arising within the retained clinical comparisons. Biomechanics requires a different qualification: it is an autonomous scientific discipline, not a component of rehabilitation medicine, and our decision not to include it among the five clinical comparisons reflects the scope of that comparison—which concerns clinical specialties encountered at the point of patient care—rather than any judgment about its standing or its centrality to the study of gait. Its relationship to this framework is addressed in Section 4.

The three-layer structure was not adopted from any single prior framework. It emerged from the organization of a large set of clinical and conceptual observations, which converged on three recurring dimensions along which walking-related presentations could be sorted. Established frameworks—the International Classification of Functioning, Disability and Health, and the biopsychosocial model among them—were consulted after the structure had taken shape, as points of comparison rather than as templates from which it was derived; the relationship of the resulting model to those frameworks is examined in Section 4.1.

One feature of this synthesis shapes the character of the framework that resulted. The categories did not begin as abstractions about walkers as a population; they were built upward from encounters with individual patients. What organized the emerging structure was a recurring sequence at the level of the single patient: the concern to preserve that person’s walking, the external conditions that made walking possible or impossible for them, and the significance their walking held in their particular life. The three layers are, in this sense, a generalization of a pattern that appeared first at the level of the individual clinical encounter rather than a schema imposed upon it.

The decision to treat meaning as a layer coordinate with the somatic and environmental, rather than as a subordinate psychological factor, rests on a specific clinical observation. Among patients whose bodily function and environmental circumstances were closely comparable, the social and personal significance a patient attached to walking tracked with marked differences in treatment response and in the durability of motivation. Such differences were not readily accounted for by somatic-layer or environmental-layer variables alone. This persistent explanatory gap—consistent enough to demand a structural place in the model rather than an occasional caveat—is what warranted establishing meaning as an independent layer.

The three coupling mechanisms were derived through a combination of inductive abstraction from clinical cases, comparison against the literature, and iterative discussion among the authors; the model and its figure passed through approximately 10 revisions before reaching the form presented here. Their number is not arbitrary. Given three layers, the interactions of principal clinical interest organize into three directional pathways of transduction—body reshaping the effective environment, environment mediating the routes of meaning, and meaning regulating somatic engagement—each of which corresponded to a pattern recurrently observed in practice. The couplings are presented not as an exhaustive account of every possible interaction but as the three whose clinical signal was strongest and most reproducible.

The synthesis was shaped throughout by the range of disciplines represented among the authors, whose expertise spans podiatry, rehabilitation and gait analysis, orthopedic and sports medicine, the internal management of diabetic foot disease, vascular surgery, prosthetics and orthotics, dermatological and plantar mechanoreceptor function, and the management of intractable wounds, alongside a public health orientation toward prevention at the population level. This range is not incidental to the framework; the layers and their couplings were tested against the clinical realities each specialty encounters. That testing drew on a substantial institutional caseload—on the order of two hundred thousand patient encounters and six thousand surgical procedures documented in clinical records, and collaborative development work with approximately eighty companies—which supplied the range of presentations against which the model’s categories were repeatedly checked.

Two features of this approach define its evidentiary status. The framework is grounded in clinical observation and organized through theory synthesis; it is not, and does not claim to be, validated by systematic evidence or prospective data. Existing frameworks were engaged as comparators rather than as foundations, which locates the model’s novelty in its integrative architecture rather than in any single borrowed construct. The limitations that follow from this status—including the absence of systematic literature synthesis and the reliance on single-institution clinical observation—are considered explicitly in the Limitations section below.

## 3 The three-layer model

The field integrates three coupled layers: somatic, environmental, and meaning. We describe each below, then articulate the coupling mechanisms by which they interact (Section 3.4).

Before detailing each layer, we specify the criterion by which a given determinant of walking is assigned to one layer rather than another. A determinant belongs to the somatic layer if it operates through the body’s internal physiological, neurological, cognitive, or affective machinery—that is, if its primary mechanism of action is located within the person, even when its trigger originates elsewhere. A determinant belongs to the environmental layer if it operates through the physical or technological structure that mediates a person’s interaction with the world—that is, if it is a property of the space, device, or infrastructure a person moves through or with, independent of any single individual’s internal state. A determinant belongs to the meaning layer if it operates through the significance walking holds for a person’s roles, relationships, and self-understanding—that is, if its primary mechanism of action is neither physiological nor infrastructural but symbolic and social.

**Figure 1:**
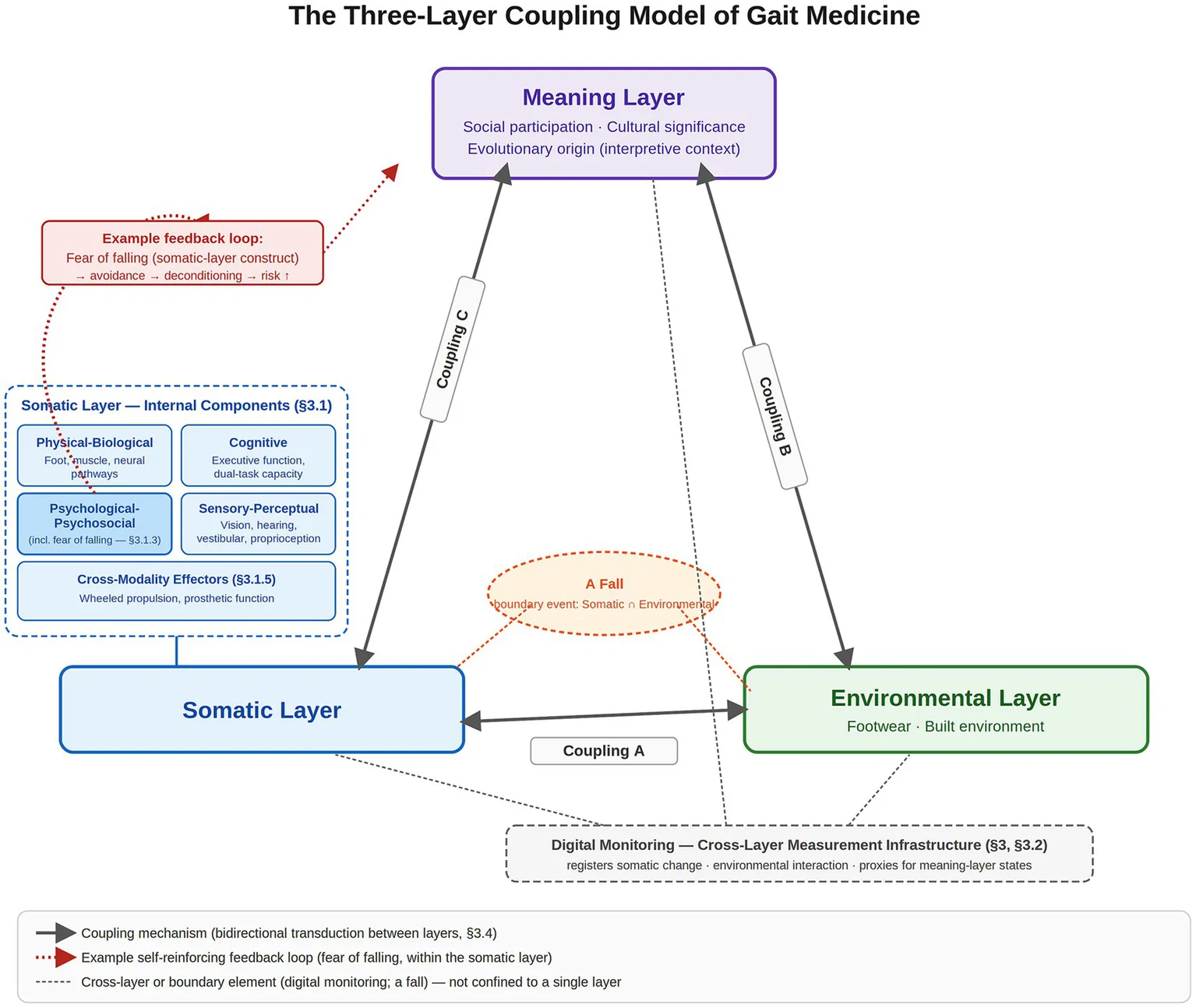
The three-layer coupling model of Gait Medicine. The somatic, environmental, and meaning layers are coupled subsystems rather than parallel domains. Three bidirectional coupling mechanisms (A, B, C) describe how dysfunction in one layer propagates to the others. The example feedback loop (red bar) illustrates how a somatic-layer construct (fear of falling) can drive environmental and further somatic outcomes through self-reinforcing cycles, consistent with the layer-assignment criteria specified in Section 3.

This criterion also clarifies the classification of specific constructs discussed below. Fear of falling, for instance, is triggered by an external event but its mechanism of action is internal: it operates through altered psychomotor drive, attentional narrowing, and avoidance behavior that reshape gait directly, in the same manner that depression does (Section 3.1). Fear of falling is therefore classified within the somatic layer throughout this paper, including in Figure 1, where it appears as an example of a somatic-layer construct whose downstream effects propagate into the environmental and meaning layers via the coupling mechanisms described in Section 3.4. The hazard that precipitates a fall—an icy patch, a broken curb, poor lighting—is itself an environmental-layer property, since it is a feature of the infrastructure independent of any individual who encounters it. A fall, by contrast, is an event at the interface of the somatic and environmental layers: it results from the interaction between a person’s balance capacity and that environmental hazard, and cannot be classified as belonging to either layer alone. The fear a fall subsequently produces is a somatic-layer response, since its mechanism of action—altered motivational drive and avoidance behavior—operates within the person regardless of where the precipitating event is classified.

Digital monitoring technology presents a different classification question. Wearable and smartphone-based gait monitoring do not themselves constitute a determinant of walking in the way that footwear or sidewalk design do; a sensor does not change how a person walks. What these technologies do is render visible, continuously and in free-living conditions, phenomena that would otherwise be assessed only intermittently and under laboratory constraints. We therefore treat digital monitoring not as a determinant within any single layer, but as measurement infrastructure that cuts across all three—capable of registering somatic change (gait speed, variability), environmental interaction (routes taken, terrain encountered), and, prospectively, proxies for meaning-layer states (outdoor excursion frequency as a marker of social participation). Its discussion in Section 3.2 reflects its practical deployment alongside other environmental-layer tools, but its analytic status is cross-layer rather than environmental.

Evolutionary origin, discussed in Section 3.3, occupies a distinct position within the meaning layer. Unlike social participation or cultural practice, evolutionary origin is not a modifiable clinical target; no intervention alters the anatomical costs of bipedalism. Its inclusion in the meaning layer is justified not because it is actionable in the way fear of falling or footwear access are actionable, but because it supplies the deepest explanatory layer for why walking carries the biomechanical vulnerabilities it does, and why its loss carries the weight it does for those who experience it. It functions in this model as interpretive context for the other layers rather than as an independent point of clinical intervention—a distinction we make explicit here to avoid conflating explanatory depth with actionability.

These criteria are not offered as a claim that every determinant of walking sorts cleanly and permanently into a single layer. Some, like a fall itself or digital monitoring, are better understood as occurring at the boundary between layers or cutting across them by design. The criteria are offered instead as a test that can be applied consistently: what is the primary mechanism of action, and where is it located? Applying this test throughout the sections that follow is what distinguishes the three-layer model from an illustrative taxonomy and moves it toward a specified theoretical structure.

### 3.1 The somatic layer

This layer covers the internal physiological, neurological, sensory, and affective machinery through which a person walks or otherwise achieves autonomous mobility—the dimension most familiar to readers of gait research, and the layer in which the criterion specified above locates any determinant whose primary mechanism of action lies within the person.

The somatic layer is not a single undifferentiated category. We distinguish three components: physical-biological, cognitive, and psychological-psychosocial. These components are frequently discussed together in gait research, but they influence walking through substantially different pathways, and conflating them obscures rather than clarifies the mechanisms available for clinical intervention.

A terminological clarification is warranted. “Somatic” is used here in its broader sense—pertaining to the person as an embodied whole—rather than in the narrower sense of bodily tissue or physiological process. The layer is defined by the location of a determinant’s mechanism of action, not by its ontological character: what unites foot structure, executive function, and fear of falling within this layer is that each operates through processes internal to the person, in contrast to determinants that operate through infrastructure (environmental) or through symbolic and social significance (meaning). Psychosocial variables such as self-efficacy and social isolation are included here because their mechanism of action on gait is intrapersonal—altered motivational drive, avoidance behavior—even though their origin is social. Their social origin is addressed within the meaning layer; their intrapersonal operation is addressed here. We retain the term “somatic” because the alternative—naming the layer by enumeration of its contents—would obscure the single criterion that defines it.

#### 3.1.1 Physical-biological contributions

The physical-biological component encompasses foot structure, the musculoskeletal system, and the peripheral and central neural pathways that execute movement. Habitual loading patterns and footwear environment shape foot morphology in ways that influence gait mechanics across the lifespan (14, 15), alongside the coordinated activity of central and peripheral neural pathways and sufficient muscular strength. Sensory contributions, which operate through partially independent pathways, are addressed separately in Section 3.1.4. This component also includes the alternative somatic effectors—upper limb propulsion, residual limb function—relevant to non-ambulatory mobility, detailed in Section 3.1.5.

#### 3.1.2 Cognitive contributions

Cognitive contributions to gait extend well beyond age-related decline narrowly construed. Executive function, attention, and dual-task processing capacity determine a person’s ability to navigate complex or changing environments while walking, and their disruption—whether through normal aging, intellectual disability, developmental disorders, acquired brain injury, or neurodegenerative disease—affects gait through mechanisms distinct from those governing muscle strength or joint mechanics. A systematic review of prospective studies found that dual-task gait performance—the decline in walking speed or stability when a concurrent cognitive task is introduced—predicts progression from normal cognition to mild cognitive impairment and from mild cognitive impairment to dementia, indicating that gait under cognitive load carries diagnostic information not captured by gait assessed in isolation (16). This relationship is bidirectional in clinical significance: a systematic review and meta-analysis of instrumented gait and balance assessment found that patients with mild cognitive impairment show measurable deficits in gait and balance parameters compared to cognitively intact peers, with the magnitude of impairment increasing under dual-task conditions (17). Walking is therefore rarely a purely automatic motor behavior; it typically competes for cognitive resources with concurrent tasks—conversation, environmental scanning, decision-making at intersections—and the capacity to allocate attention across these demands is itself a determinant of safe and effective mobility, particularly in populations with reduced cognitive reserve.

#### 3.1.3 Psychological-psychosocial contributions

Psychological and psychosocial factors constitute a third component, operating through pathways distinct from both physical-biological capacity and cognitive processing. Depression and gait speed are bidirectionally associated: depressive symptoms diminish psychomotor drive and predict measurably slower walking over time, just as slower walking predicts subsequent depressive symptoms (18). Fear of falling, a construct distinct from actual fall history, triggers avoidance of walking that accelerates deconditioning in a self-reinforcing cycle (7, 8); as specified above, its mechanism of action—altered motivational drive and avoidance behavior—locates it within this component of the somatic layer regardless of whether its trigger was an external event. Self-efficacy, social isolation, and caregiver support likewise influence gait through psychosocial pathways that are mechanistically independent of cognitive impairment: a person can retain full executive function while nonetheless avoiding walking out of fear, low self-efficacy, or diminished motivation following the loss of a walking companion or destination.

These three components are not offered as a rigid taxonomy. Many clinical presentations implicate more than one simultaneously—a patient with mild cognitive impairment who is also depressed following a fall presents somatic-layer dysfunction operating through cognitive and psychological-psychosocial pathways at once. The distinction is offered because clinical intervention differs by pathway: cognitive contributions call for dual-task training and environmental simplification, while psychological-psychosocial contributions call for psychotherapeutic and motivational approaches that address fear and self-efficacy directly. Treating the somatic layer as undifferentiated risks defaulting to physical-biological intervention—strength training, orthotic prescription—when the primary mechanism driving a patient’s gait decline lies elsewhere.

#### 3.1.4 Sensory-perceptual contributions to gait

The somatic layer, as defined above, extends beyond musculoskeletal structure and neuromuscular control to include the sensory systems that inform and calibrate walking in real time. Vision, hearing, vestibular function, and proprioception each contribute distinct and partially independent input to gait control, and their age-related decline constitutes a determinant of walking ability that is underrepresented in conventional gait assessment.

The layer assignment of sensory function warrants explicit justification, since sensory systems might appear to straddle the somatic and environmental layers. Applying the criterion specified in Section 3 resolves this. Sensory reception is a somatic-layer function: its mechanism of action—transduction of physical stimuli into neural signals, and the integration of those signals into postural and locomotor control—operates entirely within the person, and degrades or recovers according to internal physiological state rather than environmental configuration. What sensory systems process is environmental information; the systems that do the processing are not themselves environmental. The apparent straddling reflects not a misclassification but a coupling: the relationship between sensory capacity and environmental complexity is an instance of Coupling A described in Section 3.4, in which the body constitutes the effective environment. A person with intact vision and a person with severe contrast sensitivity loss, walking the same poorly lit corridor, are in different effective environments despite identical physical surroundings. Recognizing this as coupling rather than as grounds for a separate sensory layer preserves the model’s parsimony while capturing what a separate layer would be introduced to capture.

Visual input governs obstacle detection, anticipatory postural adjustment, and spatial orientation during locomotion. In a large community-based cohort of Japanese adults aged 70 years and older, visual impairment was independently associated with slower gait speed and reduced single-leg stance time after multivariate adjustment (19). Hearing loss, similarly, extends beyond communication to functional mobility: older adults with age-related hearing loss show measurably poorer performance on the Short Physical Performance Battery and greater gait variability under dual-task conditions than matched controls with normal hearing (20). A systematic review of cognitive-motor interference in hearing-impaired older adults further indicates that auditory decline compromises gait and balance in part through its cognitive load on attention and dual-task processing (21). Population-based data on physical functioning corroborate this pattern: hearing impairment is independently associated with reduced 6-min walk distance and poorer balance test performance in community-dwelling older adults in the United States (22).

Vestibular function contributes a further, distinct input. Age-related vestibular decline is associated with impaired balance and elevated fall risk, a relationship formalized in a National Institute on Aging consensus report on aging, vestibular function, and balance (23). At the level of gait kinematics, reduced semicircular canal function is associated with longer, slower steps in otherwise healthy adults, indicating that vestibular input shapes the spatial and temporal organization of walking independently of musculoskeletal capacity (24).

Proprioceptive and broader somatosensory input complete this picture. Proprioception—the awareness of the mechanical and spatial state of the body and its musculoskeletal parts—has traditionally been assessed through tests of limb position or movement detection, but recent work argues that such tests capture only low-level proprioceptive judgments and fail to account for the higher-level spatial judgments that gait and balance actually demand, a limitation with direct implications for how proprioceptive contributions to walking should be evaluated clinically (25). At the interventional level, a systematic review of seventy studies found that proprioceptive training produced comparable gains in both proprioceptive function and motor performance, with effects generalizing across neurological and musculoskeletal conditions (26). A randomized controlled trial of proprioceptive exercise in institutionalized older adults similarly demonstrated significant improvements in Timed Up and Go performance, the Tinetti scale, and single-leg stance following a structured proprioceptive training program, providing direct evidence that proprioceptive input is not merely correlated with gait quality but causally implicated in it (27). A broader systematic review of four major sensory impairments—vision, hearing, smell, and taste—found consistent associations between sensory decline and sarcopenia-related outcomes including slow gait speed and low handgrip strength across thirteen population-based studies comprising over 68,000 community-dwelling adults (28).

These sensory modalities rarely decline in isolation. In a longitudinal cohort of adults aged 70–79 years, dual sensory impairment—the combination of vision and hearing loss—predicted incident mobility difficulty and difficulty with activities of daily living over 6 years of follow-up, with substantially greater risk than either impairment alone (29). This finding is consistent with the coupling logic introduced in Section 3.4: sensory decline does not act as an isolated somatic deficit but compounds with fear of falling, reduced environmental tolerance, and diminished walking confidence to produce a magnitude of functional decline that single-modality assessment would be expected to underestimate.

Clinically, this evidence base argues against treating sensory screening as peripheral to gait assessment. A Gait Clinic model consistent with the framework proposed in Section 6.1 would include vision and hearing screening, and where resources permit, vestibular and proprioceptive assessment, as standard components of the somatic evaluation—not as referrals contingent on unrelated complaints.

#### 3.1.5 Somatic contributions across mobility modalities

The somatic layer, as framed above, has been described primarily in terms of the neuromuscular and sensory systems that support walking. This framing risks reproducing the very narrowing the field seeks to avoid. Where autonomous mobility is achieved through wheeled mobility, prosthetic limbs, or assistive technology rather than through walking, the somatic layer does not disappear—it is reorganized around different effectors and different sensorimotor demands.

For wheelchair users, propulsion strength, trunk control, and upper limb biomechanics occupy the functional position that gait mechanics occupy for ambulatory patients. The factors that determine whether powered wheelchair use translates into functional independence are themselves multifactorial, spanning individual adjustment, functional performance, and device-environment fit (30). This is structurally analogous to the somatic-environmental coupling described in Section 3.4: wheelchair mobility, like walking, is not reducible to device capability alone but emerges from the interaction between bodily function and device design.

For prosthesis users, mobility, satisfaction, and quality of life are strongly and consistently associated, a relationship documented across long-term follow-up in dysvascular and diabetic amputee populations (31). Structured intervention can shift this relationship directly: a pilot study of a dedicated mobility clinic for people with lower limb amputation found measurable improvements in self-reported mobility and quality of life following a short, intensive program (32). This finding is directly relevant to the Gait Clinic model proposed in Section 6.1—it suggests that an integrative, multidisciplinary clinical structure organized around mobility, rather than around a single modality of movement, produces measurable benefit regardless of whether the mobility in question is walking-based.

Beyond the individual device, assistive technology for mobility more broadly—powered wheelchairs, robotic gait assistance, environmental control interfaces—has been framed within a rehabilitation engineering literature that predates but anticipates the coupling logic proposed here: assistive devices function not as isolated somatic replacements but as components of an integrated system linking user capability, device design, and environmental demand (33). Scoping work on power mobility devices specifically has linked their use to occupational participation and quality of life in people with chronic disease, while identifying environmental and service-provision factors as significant moderators of outcome—directly paralleling the environmental-layer coupling this model proposes for walking (34).

Emerging transportation technology extends this picture further. Focus group research with people with disabilities, caregivers, and transportation designers has identified both the promise and the unresolved design challenges of accessible autonomous vehicles, including infrastructure, safety, and interface requirements specific to users of powered mobility devices (35). This work underscores a broader point: autonomous mobility, as this field defines it, is not achieved by walking alone or by any single technology, but by the fit between an individual’s somatic capacity—however that capacity is organized—and the systems, environmental and technological, that support it.

The clinical implication is direct. A Gait Clinic model that excludes wheelchair users, prosthesis users, and users of powered mobility devices would not merely be incomplete; it would contradict the field’s own foundational claim that autonomous mobility, not walking per se, is the organizing concept.

### 3.2 The environmental layer

Walking does not happen in a vacuum. Much of clinical gait assessment, however, proceeds as though it does—evaluating patients in hallways and laboratories, under controlled conditions, divorced from the environments in which they actually walk.

The most immediate environmental factor is footwear. Populations that habitually walk barefoot develop distinct foot morphology and gait kinematics compared to those that wear shoes, with implications for musculoskeletal health that extend well beyond the foot itself (14, 15). At the scale of the street, the built environment—sidewalk width, curb height, signal timing, street lighting—directly determines whether a particular person can walk safely in a particular place (36). Zoom further out and land-use patterns shape walking at the population level: neighborhoods designed for walkability show lower rates of obesity and cardiovascular disease (37, 38).

Digital measurement technologies are discussed here for practical rather than analytic reasons. As specified in Section 3, they are not environmental-layer determinants but cross-layer measurement infrastructure; they are presented alongside environmental-layer content because that is where they are typically deployed in practice. Wearable accelerometers and smartphone-based gait monitoring now allow continuous, free-living assessment of gait parameters that were previously measurable only under laboratory conditions, as demonstrated in neurodegenerative disease cohorts (39, 40), with accelerometer-based approaches extending to broader aging populations (41). These devices do more than refine existing measurements. They reframe the clinical question—from “how does this patient walk in my clinic?” to “how does this patient actually move through their daily world?” That shift in perspective is not incremental. It is foundational.

### 3.3 The meaning layer

This layer departs most sharply from how medicine usually thinks about walking. We believe it is also the most essential.

Walking is a vehicle for social participation. The ability to leave one’s home, visit neighbors, access a place of worship, or commute to work depends, for most of the world’s population, on the ability to walk (9, 10, 42). When walking ability declines, what follows is not merely reduced locomotion. It is a cascade—social withdrawal, loss of occupational and familial roles, depression, accelerated functional decline. Within the ICF framework of body function, activity, and participation (43), loss of walking ability is associated with social isolation, diminished occupational and familial roles, and increased care dependency. Gait Medicine takes this structure seriously. Measuring gait speed without assessing social participation is like measuring blood pressure without asking about end-organ damage.

Walking also carries cultural meaning that clinical medicine almost never engages with. Anthropological scholarship has explored how the world is perceived and constructed through the feet—how gait is culturally shaped and, reciprocally, how walking shapes cultural identity (44). The history of walking as pilgrimage, as promenade, as political protest, as meditative practice, assigns to the act of walking a significance that exceeds any biomechanical description (45). These observations are not ornamental. They explain why two patients with identical gait speed may experience their walking limitation in radically different ways, and why effective clinical intervention must address meaning alongside mechanics.

At the deepest level of this layer sits evolutionary origin. Bipedal locomotion is among the most distinctive traits separating Homo from other primates (11). The energetic advantages it conferred were substantial, and the anatomical costs—lumbar lordosis, a redesigned pelvis, vulnerable knees—are the evolutionary price of upright posture. Many of the musculoskeletal conditions clinicians treat daily are, in a sense, the sequelae of this bargain. A medical field organized around walking that fails to account for this evolutionary context is incomplete in a fundamental way.

### 3.4 Layer interactions

The three layers described above are not parallel domains but coupled subsystems. Treating them merely as a taxonomy risks obscuring the central clinical insight: walking deteriorates not because one layer fails in isolation, but because dysfunction transduces across layer boundaries. Three coupling mechanisms deserve explicit articulation (Figure 1).

Coupling A—The body reshapes the effective environment. A patient with forefoot pain does not merely walk more slowly; her tolerance for sidewalk irregularities collapses, effectively shrinking her walkable neighborhood without any physical change to that neighborhood. The environment is not a fixed external stage; it is constituted, in part, by the body that traverses it. Conversely, a built environment that demands sustained postural control selectively recruits and conditions the somatic system. Body and environment are mutually constructing.

Coupling B—The environment mediates the routes of meaning. When a redesigned intersection makes the path to a place of worship impassable, what is lost is not only access but the embodied practice through which walking acquired significance for that person. Environmental change can sever the routes by which walking carries meaning, even when physical capacity is preserved. Reciprocally, when meaning-laden destinations exist, the environment becomes legible as walkable in ways that walkability indices alone fail to capture.

Coupling C—Meaning regulates somatic engagement with walking. When walking no longer serves visible purposes—visiting, working, worshipping, belonging—its motivational substrate is depleted. Reduced walking produces deconditioning, which compounds with depressive symptoms and fear of falling to drive measurable gait deterioration. Meaning-deprivation is not metaphorical; we propose that it functions as an upstream input to somatic outcomes, one that Section 3.4 specifies as a testable prediction rather than an established mechanism.

These couplings generate feedback loops with clinical consequence. The fear-of-falling cycle—perceived risk leading to avoidance, deconditioning, actual risk increase, and reinforced fear—is one instance of a more general structure in which each layer’s state is partly determined by, and partly determines, the others. This structure has direct implications for intervention: targeting any single layer in isolation may be insufficient, while interventions that address cross-layer dynamics could plausibly yield substantial gains.

The coupling model yields a falsifiable prediction. Here we specify it with the precision necessary for empirical testing: the mechanisms by which layers interact, the interventions to be compared, the outcomes to be measured, and the timescale over which effects should appear.

Mechanisms. Each coupling described above (A, B, C) specifies a distinct pathway of transduction. Coupling A predicts that somatic intervention (e.g., orthotic correction of forefoot pain) should measurably increase a patient’s tolerance for environmental irregularity—operationalized as the range of terrain types negotiated without self-reported difficulty—independent of any change to the environment itself. Coupling B predicts that environmental intervention (e.g., restoring a walkable route to a meaning-laden destination) should increase engagement with that destination beyond what would be predicted from the physical accessibility change alone, because the route’s restoration reactivates a previously severed meaning pathway. Coupling C predicts that meaning-layer intervention (e.g., reconnecting a patient with a valued destination or social role) should produce measurable downstream improvement in somatic gait parameters—gait speed, step variability—that exceeds what physical therapy alone produces in matched patients, because restored motivational salience increases both walking frequency and postural engagement during walking.

Interventions to be compared. A prospective trial testing the model’s central claim would randomize patients with comparable baseline gait impairment to one of three arms. Arm 1 receives a single-layer somatic intervention alone: orthotic prescription, strength training, and fear-of-falling-focused psychotherapy—all three of which, under the criterion specified in Section 3, are somatic-layer interventions addressing different components of that layer.

Arms 2 and 3 receive an identical set of components spanning all three layers: the somatic package described above; an environmental component consisting of a structured walkability audit and route-modification plan for the patient’s neighborhood; and a meaning-layer component consisting of structured destination-directed re-engagement—the identification, with the patient, of destinations and social roles that walking previously served, and a graded plan for restoring walking to those specific purposes. This last component is distinct in mechanism from fear-of-falling psychotherapy: it targets motivational salience and the restoration of purpose rather than the reduction of anxiety, and it is delivered independently of the psychotherapeutic component.

The two arms differ only in delivery. In Arm 2, the three components are delivered independently: each clinician works to their own assessment and plan, with no structured mechanism for exchanging findings or aligning goals. In Arm 3, the same components at the same intensity and dosage are delivered through an explicitly coordinated, coupling-informed protocol: a shared assessment, joint goal-setting across the three domains, and scheduled case conferences at which the clinicians examine how change in each layer is affecting the others—for instance, whether restored access to a valued destination is reducing avoidance behavior, or whether orthotic correction has widened the range of terrain the patient will attempt.

Because Arms 2 and 3 receive equivalent components, any superiority of Arm 3 is attributable to coordination rather than to the addition of intervention content. This is the comparison on which the model’s central claim stands or falls.

Outcomes. Consistent with the mobility facets introduced in Section 4 (46), outcomes should be measured across all three facets rather than gait speed alone. Locomotor capacity is captured by instrumented gait speed and variability, consistent with existing validated protocols (1). Actual mobility is captured by life-space assessment, using an instrument such as the Life-Space Assessment scale, which quantifies the frequency and independence of movement across concentric zones from bedroom to beyond one’s town (42). Perceived mobility and meaning-layer engagement are captured by walking-specific self-efficacy measures and a structured measure of destination-directed outdoor excursion frequency, distinguishing purposeful trips (to a place of worship, a social gathering, a family member’s home) from undirected ambulation. The model predicts that arm 3 will show superior improvement specifically on life-space and self-efficacy measures, not merely on gait speed—a pattern that would be difficult to explain without invoking cross-layer coupling.

Timescale. Somatic-layer change following orthotic and strength intervention is typically measurable within 6 weeks; meaning-layer change, operationalized as shifts in outdoor excursion frequency and self-efficacy, requires longer observation, as behavioral patterns of avoidance or re-engagement develop over months rather than weeks. We therefore specify primary outcome assessment at 6 months, with a secondary assessment at 12 months to test whether between-arm differences persist, narrow, or widen—a pattern that would itself provide evidence about whether coupling effects are transient or self-sustaining once meaning-layer engagement is restored.

One scope condition should be stated explicitly. The design specified here applies to patients whose primary mobility modality is walking, and its locomotor-capacity outcomes—gait speed, step variability—are modality-specific. Testing the coupling model in populations whose autonomous mobility is organized around wheeled propulsion or prosthetic use would require modality-appropriate substitutes at that level: propulsion efficiency, transfer independence, or prosthetic wear time in place of gait parameters. The life-space and self-efficacy measures, by contrast, are modality-neutral and would transfer directly. Whether the coupling model’s predictions hold across mobility modalities is itself an empirical question this framework generates rather than one it presupposes.

This specification does not eliminate the possibility of disconfirmation. If arm 3 fails to outperform arm 2 on life-space and self-efficacy measures at 6 months, the central coupling claim—that addressing layer interactions produces effects beyond the sum of single-layer interventions—would be substantially weakened. The model is falsifiable in a strict sense: it predicts a specific, measurable pattern of results that would not be expected under a model in which the three layers operate independently.

## 4 Distinguishing Gait Medicine from adjacent fields

Claiming that a new field occupies unique conceptual space is easy. Demonstrating it demands specificity. We compare Gait Medicine to five adjacent disciplines.

Rehabilitation medicine has developed rigorous, neuroplasticity-informed approaches to gait recovery after stroke and other neurological injury, integrating classical gait training, robotic devices, and brain-computer interfaces within a coherent clinical program (4). Its scope extends well beyond recovery from a discrete injury, encompassing sarcopenia, deconditioning, and other age- and disuse-related decline for which no single triggering event exists. Gait Medicine does not compete with this body of work. The difference is not one of scope but of organizing logic: within rehabilitation medicine, gait is typically addressed as an outcome domain nested within a broader clinical program—physical medicine, geriatric care, neurological rehabilitation—organized around the patient’s underlying condition. Gait Medicine inverts this structure, making walking itself the axis around which the relevant clinical, environmental, and social considerations are organized, regardless of which underlying condition brought the patient to clinical attention.

Sports medicine optimizes movement in athletic populations. Gait Medicine is concerned with the daily walking of the entire population, with particular focus on those who are losing or at risk of losing this ability—older adults, people with chronic conditions, and people with disabilities.

A distinction is necessary here. Sports medicine, as compared above, is a clinical specialty. Sport and exercise sciences—encompassing biomechanics, motor control, motor development, exercise physiology, adapted physical activity, and physical activity and health research—constitute a set of autonomous scientific disciplines whose contribution to the understanding of gait and mobility is foundational. Much of what is known about the mechanics of the walking step, the neural control of locomotion, and the development and degradation of locomotor patterns was established within these fields. Gait Medicine draws on that knowledge and makes no claim to disciplinary ownership of gait as an object of study.

What Gait Medicine contributes is distinct in kind rather than lesser in degree. Biomechanics explains how a walking step is produced. Motor control explains how it is regulated. Neither specifies who is responsible when forefoot pathology, an unwalkable intersection, and the loss of a valued destination converge in a single patient, nor how those determinants should be assessed together, nor what follows clinically when they are. That question—the organization of clinical attention around walking as its axis—belongs to no existing discipline, and answering it requires a form of integration that the component sciences were not designed to provide. Gait Medicine occupies that position: the clinical and translational component of a broader interdisciplinary gait and mobility science, with a role within it that is its own.

Podiatry centers on the foot and ankle. Gait Medicine starts at the foot but integrates upward through the nervous system and cognition, and outward through footwear, urban infrastructure, social participation, and cultural meaning. The foot is one part. Walking is the system.

Public health addresses population-level determinants of health but lacks a systematic framework organized specifically around walking. Gait Medicine offers a vertical structure—from the biomechanics of a single step to the design of a city block—unified by one organizing principle.

Geriatrics has produced comprehensive clinical approaches to gait disturbance in older adults, integrating neurological, musculoskeletal, and orthostatic contributions into structured diagnostic and management frameworks, and gait speed is itself a well-established component of comprehensive geriatric assessment (6). Gait Medicine draws on this body of work but is not age-restricted in the way geriatrics is by definition. A child with cerebral palsy, a 35-year-old with multiple sclerosis, and a 70-year-old with diabetic neuropathy all fall within its scope; the organizing axis is walking ability across the lifespan, not age itself. The structural difference lies in the organizing category rather than in the thoroughness of attention to gait: geriatrics organizes its assessment of walking around the aging process, within a framework that also addresses cognition, polypharmacy, and multimorbidity; Gait Medicine organizes assessment around walking itself, extending that same axis to patients for whom age is not the relevant organizing category. This is a difference in what anchors the clinical framework, not a claim that either approach attends to gait less rigorously.

The contribution, then, is architectural. Gait Medicine uses one human activity—walking—as the organizing axis around which clinical medicine, public health, urban engineering, social science, and the humanities are vertically integrated. Adjacent fields, as demonstrated above, treat walking with genuine rigor within their own scope. Gait Medicine treats it as the organizing axis itself.

### 4.1 Positioning relative to existing conceptual frameworks

Gait Medicine’s three-layer model does not emerge in a conceptual vacuum. The World Health Organization’s International Classification of Functioning, Disability and Health (ICF) has, for two decades, provided the dominant framework for describing functioning in rehabilitation and gerontology, distinguishing body functions and structures, activities and participation, and environmental and personal contextual factors (43). More recently, the WHO’s healthy aging agenda has generated a complementary framework specific to mobility, proposing that mobility be measured across three distinct facets—perceived mobility (“what can you do”), actual mobility (“what you actually do”), and locomotor capacity (“what could you do”)—each rooted in one of three WHO-endorsed components of healthy aging: functional ability, intrinsic capacity, and environment (46).

The correspondence between this unified mobility framework and the three-layer model proposed here is substantial but not exact, and the difference is instructive. Locomotor capacity corresponds closely to the somatic layer: both concern what the body is physiologically capable of, independent of context. Actual mobility corresponds to the joint operation of the somatic and environmental layers: what a person does in the world reflects the interaction between bodily capacity and environmental affordance, not capacity alone. Perceived mobility maps most closely onto the meaning layer, though imperfectly—self-reported difficulty captures something adjacent to, but not identical with, the significance walking holds for social participation and identity that the meaning layer addresses.

The divergence is where Gait Medicine’s contribution becomes visible. The unified mobility framework, like the ICF before it, is architecturally a taxonomy: it specifies what should be measured and how those measurements relate to broader constructs of health, but it does not specify mechanisms by which a change in one facet produces a change in another. Gait Medicine’s coupling model (Section 3.4) is an attempt to specify exactly this: not merely that somatic, environmental, and meaning-layer factors are related, but how dysfunction in one transduces into dysfunction in another, generating testable predictions about which combinations of intervention should outperform single-domain approaches. In this sense, Gait Medicine does not compete with the ICF or the unified mobility framework; it proposes a coupling logic that could, in principle, be layered onto either.

## 5 Anticipated objections

Three objections deserve preemptive response.

That Gait Medicine is merely an assemblage of existing knowledge, not an independent field. This objection has force precisely because the component knowledge is genuinely substantial: rehabilitation medicine, neurology, and geriatrics each maintain rigorous, well-developed literatures on gait (4–6). Gait Medicine does not contest the quality of this work, nor does it propose to duplicate it. The identical objection was raised against geriatrics in the mid-twentieth century, when internal medicine, neurology, and psychiatry each already addressed aging-related decline, and against palliative medicine more recently, when oncology and pain management already existed. In neither case was the objection answered by new empirical discoveries within the component disciplines. It was answered by demonstrating that integration around a different organizing axis reveals phenomena invisible to any single component discipline—polypharmacy cascades in geriatrics, total pain in palliative care. Gait Medicine makes a parallel claim. The interaction between urban crossing design and forefoot pathology, or between cultural attitudes toward walking and an individual’s threshold for seeking clinical help, is not unknown to any single discipline in isolation, but becomes clinically actionable only when walking is made the organizing principle around which the relevant disciplines are coordinated. The integration, not the underlying knowledge, is the intellectual contribution.

That centering a field on walking excludes those who cannot walk. We have addressed this above. The superordinate concept is autonomous mobility; walking is its most prevalent form. The same structural criticism could in principle be directed at cardiology or ophthalmology. The existence of boundary cases refines a field; it does not disqualify it.

That incorporating urban design and cultural anthropology overextends the boundaries of medicine. This objection assumes a definition of medicine that the twenty-first century has already abandoned. The Lancet series on urban design and health showed, empirically, that city planning decisions are public health decisions (38, 47). The WHO Commission on Social Determinants of Health established that health outcomes are fundamentally shaped by the social and physical conditions in which people live and age (48, 49). Gait Medicine does not annex urban planning. It identifies walking as the specific, measurable, clinically actionable point of contact between the built environment and individual health.

## 6 Clinical, public health, and research implications

If Gait Medicine is to be more than conceptual, it must translate into practice. We outline four domains.

### 6.1 The gait clinic

We envision a multidisciplinary clinical service—spanning outpatient, inpatient, and perioperative care as the clinical picture requires—organized around a single presenting concern: deteriorating walking ability. A patient who reports “I am walking less”—a complaint that currently has no obvious referral destination—would have somewhere to go.

**Figure 2:**
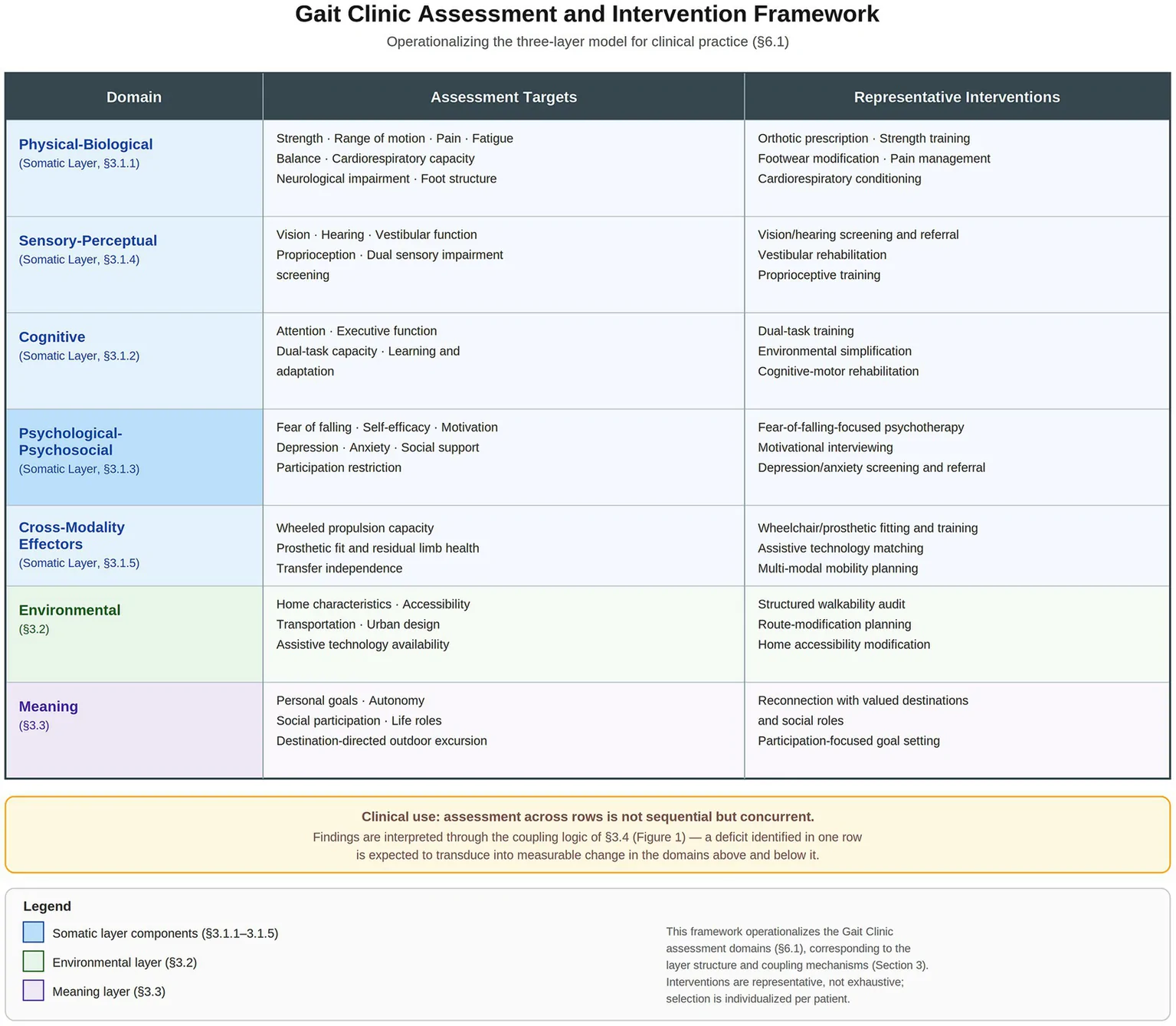
Gait Clinic assessment and intervention framework. Assessment domains and representative interventions are mapped to each layer and, within the somatic layer, to each of the components specified in Sections 3.1.1–3.1.5. Assessment across domains is concurrent rather than sequential; findings are interpreted through the coupling logic specified in Section 3.4 and depicted in Figure 1. Interventions listed are representative rather than exhaustive.

Assessment would cover gait speed and biomechanics, certainly. But it would extend to fear of falling, depression screening, footwear evaluation, and a structured assessment of neighborhood walkability. Outcomes would be measured not only in meters per second but also in social participation frequency, outdoor excursion rate, and self-efficacy around walking—capturing the meaning and environmental layers alongside the somatic. Figure 2 sets out the assessment domains and representative interventions this implies, organized by layer and, within the somatic layer, by the components specified in Section 3.1.

At Shimokitazawa Hospital in Tokyo, a specialty institution focused on foot and lower-limb medicine, the early shape of this integration is emerging. Though not yet a fully realized Gait Clinic in the sense proposed here, the institution’s structure illustrates how the model might be operationalized within an existing framework. Patients present not with isolated anatomical complaints but with compound walking difficulties shaped as much by footwear and neighborhood as by biomechanics. Preliminary clinical observation, which does not constitute systematic outcome data, is consistent with the possibility that addressing fear, footwear, and environmental context alongside tissue pathology supports engagement that isolated podiatric treatment may not achieve. This illustrates a way in which integrative gait-centered care could be organized within existing institutional structures; whether it produces superior outcomes is a separate question that rigorous prospective evaluation would be required to answer.

The Gait Clinic model outlined above is organized around walking because walking is the modality through which most patients present. This should not be mistaken for a claim that the model applies only to walking. Consider a second presentation: a 58-year-old man with a transtibial amputation secondary to peripheral vascular disease, 3 years post-amputation, who reports that he has progressively stopped using his prosthesis for anything beyond short indoor distances and now relies on a manual wheelchair for community mobility—a shift that began after a fall 6 months ago. His prosthetist notes no mechanical problem with the socket fit. What has changed is not somatic capacity in the narrow sense but the coupling between that capacity and his built and social environment: the fall, though minor and without injury, occurred on a stretch of sidewalk recently resurfaced with a grade he had not previously needed to negotiate, and it has left him reluctant to attempt uneven terrain on the prosthesis since.

Under a Gait Clinic model organized exclusively around walking, this patient’s care would likely stall at the prosthetic fitting, since the device itself is functioning as designed. Under the framework proposed here, his presentation is legible in exactly the same terms as the walking-based vignette above: somatic capacity (residual limb health, prosthetic fit, upper body strength for wheelchair propulsion, and—consistent with the criterion specified in Section 3—the fear following his fall), environmental fit (the resurfaced sidewalk, the accessibility of his routes), and meaning (the roles and destinations that motivated his prior mobility, and what their loss has meant for his sense of independence) are coupled in the same way that they are coupled for a patient whose primary mobility modality is gait. The clinical response—addressing the fall-related fear directly, auditing the specific environmental change that triggered avoidance, and evaluating whether his current mobility strategy (prosthesis for short distances, wheelchair for community distances) reflects an adaptive multi-modal solution rather than a failure requiring correction—follows the same integrative logic outlined in Section 3.1.5, regardless of whether his autonomous mobility is ultimately organized around walking, wheeled propulsion, or some combination of the two.

### 6.2 Urban policy integration

Gait Medicine supplies a clinical evidence base for walkability policy. When a municipality considers eliminating a pedestrian crossing or narrowing a sidewalk to add a traffic lane, Gait Medicine can quantify the health consequences—reduced walking, increased falls, accelerated functional decline among vulnerable populations (36–38, 50). This is not political advocacy dressed as medicine. It is applied epidemiology anchored in clinical data.

### 6.3 Digital gait biomarkers

Wearable devices now allow continuous monitoring of gait in free-living conditions, as demonstrated in neurodegenerative disease cohorts (39, 40), with accelerometer-based approaches extending to broader aging populations (41), enabling detection of deterioration well before a clinical event such as a fall. Gait Medicine provides the interpretive scaffold for these data. The clinical meaning of “gait speed declined by 0.1 m/s” is not self-evident. It requires a framework that connects the measurement to its human consequences—diminished social participation, compromised mental health, eroding autonomy. The measurement and the meaning belong together. Gait Medicine is the field that holds them together.

### 6.4 Future research directions

The conceptual framework proposed here generates a research program spanning clinical trials, implementation science, and measurement development.

The most important empirical test follows directly from the coupling model, specified in detail in Section 3.4: a three-arm trial comparing single-layer intervention, multi-layer intervention delivered without coordination, and the same multi-layer intervention delivered through an explicitly coordinated protocol, with outcomes assessed across locomotor capacity, actual mobility, and perceived mobility at 6 and 12 months. If integrative interventions outperform single-layer and uncoordinated multi-component interventions specifically on life-space and self-efficacy measures, the model’s central claim is supported; if they do not, the claim must be revised.

The meaning layer presents a distinct problem: the instruments needed to measure it do not yet exist. Existing measures of social participation are not gait-specific. Self-efficacy scales do not distinguish walking confidence from general functional confidence. Cross-cultural variation in what walking signifies—to a pilgrim, to a commuter, to someone whose mobility has just begun to decline—has barely been operationalized. This is where the field’s interdisciplinary character becomes most demanding, and most productive.

Implementation comes next. If integrative care works, can it be delivered at scale? Where? At what cost? Trials in urban academic centers, rural clinics, and resource-limited settings would identify structural barriers and clarify whether Gait Clinics can be sustained against fragmented usual care. Modeling studies projecting healthcare cost offsets—fewer falls, preserved independence, delayed institutionalization—would inform policy adoption.

Digital phenotyping adds a longer-horizon question. Wearable monitoring will eventually generate the data; the harder problem is interpretive. Which patterns of gait variability signal impending adverse outcomes, and which reflect benign day-to-day fluctuation? The three-layer model offers the scaffold within which such algorithms can be developed and validated, but the algorithms themselves remain to be built.

These directions are not exhaustive, nor are they sequential. Several can—and should—proceed in parallel.

## 7 Conclusion

Walking predicts death, dementia, depression, and disability. It is, by any reasonable definition, a vital sign. Yet it has no dedicated field, no dedicated clinic, and no research agenda that spans its full range of determinants—from foot mechanics to urban design, from the fear of falling to the evolutionary pressures that made us bipedal.

Gait Medicine does not claim to have discovered walking. The argument of this paper is not that walking has been overlooked, but that medicine has failed to organize itself around it. The evidence supports such organization. The patients need it. What is missing is the field itself.

### 7.1 Limitations

The framework presented here has several limitations that bound its current claims and define the work required to substantiate it.

First, this is a conceptual synthesis, not an empirical validation. The three-layer model and its coupling mechanisms are grounded in clinical observation and organized through theory synthesis; they have not been tested against prospective, systematically collected data. The central prediction specified in Section 3.4—that coordinated multi-layer intervention outperforms uncoordinated or single-layer alternatives—remains a hypothesis. The value of the framework at this stage lies in its capacity to organize existing knowledge and to generate testable predictions, not in demonstrated superiority of the interventions it implies.

Second, the literature synthesized here was assembled selectively rather than systematically. As set out in the Methodological Approach, we did not conduct a systematic or scoping review, and the reference base does not constitute an exhaustive survey of any of the fields it touches. This carries a specific risk: relevant frameworks, particularly in mobility science, movement science, and disability studies, may be underrepresented, and the boundaries drawn between Gait Medicine and adjacent disciplines reflect the authors’ vantage point rather than a comprehensive mapping of those fields. We have sought to mitigate this by engaging the most directly comparable existing framework explicitly (Section 4.1), but the risk of selective emphasis remains inherent to the method.

Third, the clinical observations that informed the model derive substantially from a single specialized institution. This introduces two constraints. The range of presentations, though large, reflects the case mix of a foot- and lower-limb-focused center, which may overrepresent certain pathways and underrepresent others—cognitive and neurological contributions to gait, for instance, relative to musculoskeletal ones. And because several authors are affiliated with that institution, the description of its clinical structure should be read as illustrative of how the framework can be operationalized, not as evidence that operationalizing it produces superior outcomes. The two claims are distinct, and only the former is supported here.

Fourth, the meaning layer, which we argue is essential to the framework, is also the layer for which validated measurement is least developed. Existing instruments for social participation and self-efficacy are not walking-specific, and the cross-cultural variation in what walking signifies has barely been operationalized. The framework therefore specifies an outcome domain that current instruments only partially capture, and the measurement development this requires is itself part of the research program the model implies, as outlined in Section 6.4.

Fifth, the framework’s generalization beyond ambulatory populations, though argued for on conceptual grounds, has not been worked through at the level of operational detail that walking-based presentations have received. The extension to wheeled and prosthetic mobility (Sections 3.1.5 and 6.1) establishes that the coupling logic applies in principle across mobility modalities, but the modality-specific measures and interventions this would require remain to be developed and tested.

None of these limitations is incidental; each marks a specific direction in which the framework must be developed and tested before its central claims can be regarded as established. Stating them precisely is part of specifying the theory, since a framework of this kind is strengthened, not weakened, by making explicit the conditions under which it could fail.

## Data Availability

The original contributions presented in the study are included in the article/supplementary material, further inquiries can be directed to the corresponding author.

## References

[1] Studenski S Perera S Patel K Rosano C Faulkner K Inzitari M . Gait speed and survival in older adults. JAMA. (2011) 305:50–8. doi: 10.1001/jama.2010.1923, 21205966 PMC3080184

[2] Verghese J Lipton RB Hall CB Kuslansky G Katz MJ Buschke H. Abnormality of gait as a predictor of non-Alzheimer’s dementia. N Engl J Med. (2002) 347:1761–8. doi: 10.1056/NEJMoa020441, 12456852

[3] Montero-Odasso M Schapira M Soriano ER Varela M Kaplan R Camera LA . Gait velocity as a single predictor of adverse events in healthy seniors aged 75 years and older. J Gerontol A Biol Sci Med Sci. (2005) 60:1304–9. doi: 10.1093/gerona/60.10.1304, 16282564

[4] Belda-Lois JM Mena-del Horno S Bermejo-Bosch I Moreno JC Pons JL Farina D . Rehabilitation of gait after stroke: a review towards a top-down approach. J Neuroeng Rehabil. (2011) 8:66. doi: 10.1186/1743-0003-8-66, 22165907 PMC3261106

[5] Fasano A Bloem BR. Gait disorders. Continuum (Minneap Minn). (2013) 19:1344–82. doi: 10.1212/01.CON.0000436159.33447.69, 24092293

[6] Pirker W Katzenschlager R. Gait disorders in adults and the elderly: a clinical guide. Wien Klin Wochenschr. (2017) 129:81–95. doi: 10.1007/s00508-016-1096-4, 27770207 PMC5318488

[7] Delbaere K Crombez G Vanderstraeten G Willems T Cambier D. Fear-related avoidance of activities, falls and physical frailty. A prospective community-based cohort study. Age Ageing. (2004) 33:368–73. doi: 10.1093/ageing/afh106, 15047574

[8] Rochat S Büla CJ Martin E Seematter-Bagnoud L Karmaniola A Aminian K . What is the relationship between fear of falling and gait in well-functioning older persons aged 65 to 70 years? Arch Phys Med Rehabil. (2010) 91:879–84. doi: 10.1016/j.apmr.2010.03.005, 20510978

[9] Shimada H Ishizaki T Kato M Morimoto A Tamate A Uchiyama Y . How often and how far do frail elderly people need to go outdoors to maintain functional capacity? Arch Gerontol Geriatr. (2010) 50:140–6. doi: 10.1016/j.archger.2009.02.015, 19356806

[10] Webber SC Porter MM Menec VH. Mobility in older adults: a comprehensive framework. Gerontologist. (2010) 50:443–50. doi: 10.1093/geront/gnq013, 20145017

[11] Sockol MD Raichlen DA Pontzer H. Chimpanzee locomotor energetics and the origin of human bipedalism. Proc Natl Acad Sci USA. (2007) 104:12265–9. doi: 10.1073/pnas.0703267104, 17636134 PMC1941460

[12] Walker LO Avant KC. Strategies for Theory Construction in Nursing. 6th ed. Boston: Pearson (2019).

[13] Jaakkola E. Designing conceptual articles: four approaches. AMS Rev. (2020) 10:18–26. doi: 10.1007/s13162-020-00161-0

[14] Hollander K de Villiers JE Sehner S Wegscheider K Braumann KM Venter R . Growing-up (habitually) barefoot influences the development of foot and arch morphology in children and adolescents. Sci Rep. (2017) 7:8079. doi: 10.1038/s41598-017-07868-4, 28808276 PMC5556098

[15] D'Août K Pataky TC De Clercq D Aerts P. The effects of habitual footwear use: foot shape and function in native barefoot walkers. Footwear Sci. (2009) 1:81–94. doi: 10.1080/19424280903386411

[16] Ramírez F Gutiérrez M. Dual-task gait as a predictive tool for cognitive impairment in older adults: a systematic review. Front Aging Neurosci. (2021) 13:769462. doi: 10.3389/fnagi.2021.769462, 35002676 PMC8740025

[17] Bahureksa L Najafi B Saleh A Sabbagh M Coon D Mohler MJ . The impact of mild cognitive impairment on gait and balance: a systematic review and meta-analysis of studies using instrumented assessment. Gerontology. (2017) 63:67–83. doi: 10.1159/000445831, 27172932 PMC5107359

[18] Demakakos P Cooper R Hamer M de Oliveira C Hardy R Breeze E. The bidirectional association between depressive symptoms and gait speed: evidence from the English longitudinal study of ageing (ELSA). PLoS One. (2013) 8:e68632. doi: 10.1371/journal.pone.0068632, 23874698 PMC3706406

[19] Miyata K Yoshikawa T Harano A Ueda T Ogata N. Effects of visual impairment on mobility functions in elderly: results of Fujiwara-kyo eye study. PLoS One. (2021) 16:e0244997. doi: 10.1371/journal.pone.0244997, 33513151 PMC7845963

[20] Kolasa S Magnussen LH Nilsen RM Wilhelmsen KT Goplen FK Nordahl SHG . Walking and balance in older adults with age-related hearing loss: a cross-sectional study of cases and matched controls. Gait Posture. (2024) 113:398–406. doi: 10.1016/j.gaitpost.2024.07.301, 39088930

[21] Wunderlich A Wollesen B Asamoah J Delbaere K Li K. The impact of cognitive-motor interference on balance and gait in hearing-impaired older adults: a systematic review. Eur Rev Aging Phys Act. (2024) 21:17. doi: 10.1186/s11556-024-00350-x, 38914940 PMC11194914

[22] Martinez-Amezcua P Powell D Kuo PL Reed NS Sullivan KJ Palta P . Association of age-related hearing impairment with physical functioning among community-dwelling older adults in the US. JAMA Netw Open. (2021) 4:e2113742. doi: 10.1001/jamanetworkopen.2021.13742, 34170305 PMC8233700

[23] Agrawal Y Merfeld DM Horak FB Redfern MS Manor B Westlake KP . Aging, vestibular function, and balance: proceedings of a national institute on aging/national institute on deafness and other communication disorders workshop. J Gerontol A Biol Sci Med Sci. (2020) 75:2471–80. doi: 10.1093/gerona/glaa097, 32617555 PMC7662183

[24] Anson E Pineault K Bair W Studenski S Agrawal Y. Reduced vestibular function is associated with longer, slower steps in healthy adults during normal speed walking. Gait Posture. (2019) 68:340–5. doi: 10.1016/j.gaitpost.2018.12.016, 30576978 PMC6370495

[25] Héroux ME Butler AA Robertson LS Fisher G Gandevia SC. Proprioception: a new look at an old concept. J Appl Physiol (1985). (2022) 132:811–4. doi: 10.1152/japplphysiol.00809.2021, 35142561

[26] Winter L Huang Q Sertic JVL Konczak J. The effectiveness of proprioceptive training for improving motor performance and motor dysfunction: a systematic review. Front Rehabil Sci. (2022) 3:830166. doi: 10.3389/fresc.2022.830166, 36188962 PMC9397687

[27] Espejo-Antúnez L Pérez-Mármol JM Cardero-Durán MLÁ Toledo-Marhuenda JV Albornoz-Cabello M. The effect of proprioceptive exercises on balance and physical function in institutionalized older adults: a randomized controlled trial. Arch Phys Med Rehabil. (2020) 101:1780–8. doi: 10.1016/j.apmr.2020.06.010, 32663479

[28] Ho KC Gupta P Fenwick EK Man REK Gan ATL Lamoureux EL. Association between age-related sensory impairment with sarcopenia and its related components in older adults: a systematic review. J Cachexia Sarcopenia Muscle. (2022) 13:811–23. doi: 10.1002/jcsm.12930, 35229470 PMC8977955

[29] Armstrong NM Vieira Ligo Teixeira C Gendron C Brenowitz WD Lin FR Swenor B . Associations of dual sensory impairment with incident mobility and ADL difficulty. J Am Geriatr Soc. (2022) 70:1997–2007. doi: 10.1111/jgs.17764, 35343588 PMC9283239

[30] Fishleigh L Taylor R Hale G Bowers DS. Factors that affect powered wheelchair use for an adult population: a systematic review. Disabil Rehabil Assist Technol. (2024) 19:2651–64. doi: 10.1080/17483107.2024.2304122, 38287878

[31] Wurdeman SR Stevens PM Campbell JH. Mobility analysis of AmpuTees (MAAT 6): mobility, satisfaction, and quality of life among long-term dysvascular/diabetic prosthesis users. J Prosthet Orthot. (2021) 33:161–7. doi: 10.1097/JPO.0000000000000304, 34177205 PMC8216599

[32] Anderson S Ridgewell E Dillon M. The effect of participation in a mobility clinic on self-reported mobility and quality of life in people with lower limb amputation: a pilot study. Prosthetics Orthot Int. (2020) 44:202–7. doi: 10.1177/0309364620921749, 32500815

[33] Cowan RE Fregly BJ Boninger ML Chan L Rodgers MM Reinkensmeyer DJ. Recent trends in assistive technology for mobility. J Neuroeng Rehabil. (2012) 9:20. doi: 10.1186/1743-0003-9-20, 22520500 PMC3474161

[34] Kemmis E Ashby S MacDonald-Wicks L. The impact of a power mobility device on occupational participation and quality of life for people with chronic diseases: a scoping review. Br J Occup Ther. (2021) 84:780–92. doi: 10.1177/03080226211034420

[35] Sivakanthan S Cooper R Lopes C Kulich H Deepak N Lee CD . Accessible autonomous transportation and services: a focus group study with people with disabilities and stakeholders. Disabil Rehabil Assist Technol. (2024) 19:1992–9. doi: 10.1080/17483107.2023.2242898 37548013

[36] Rosso AL Auchincloss AH Michael YL. The urban built environment and mobility in older adults: a comprehensive review. J Aging Res. (2011) 2011:816106. doi: 10.4061/2011/816106, 21766033 PMC3134204

[37] Frank LD Sallis JF Conway TL Chapman JE Saelens BE Bachman W. Many pathways from land use to health: associations between neighborhood walkability and active transportation, body mass index, and air quality. J Am Plan Assoc. (2006) 72:75–87. doi: 10.1080/01944360608976725

[38] Sallis JF Cerin E Conway TL Adams MA Frank LD Pratt M . Physical activity in relation to urban environments in 14 cities worldwide: a cross-sectional study. Lancet. (2016) 387:2207–17. doi: 10.1016/S0140-6736(15)01284-2, 27045735 PMC10833440

[39] Lipsmeier F Taylor KI Kilchenmann T Wolf D Scotland A Schjodt-Eriksen J . Evaluation of smartphone-based testing to generate exploratory outcome measures in a phase 1 Parkinson’s disease clinical trial. Mov Disord. (2018) 33:1287–97. doi: 10.1002/mds.27376, 29701258 PMC6175318

[40] Del Din S Godfrey A Mazzà C Lord S Rochester L. Free-living monitoring of Parkinson’s disease: lessons from the field. Mov Disord. (2016) 31:1293–313. doi: 10.1002/mds.26718, 27452964

[41] Mañas A Del Pozo-Cruz B Guadalupe-Grau A Marín-Puyalto J Alfaro-Acha A Rodríguez-Mañas L . Reallocating accelerometer-assessed sedentary time to light or moderate- to vigorous-intensity physical activity reduces frailty levels in older adults: an isotemporal substitution approach in the TSHA study. J Am Med Dir Assoc. (2018) 19:185.e1–6. doi: 10.1016/j.jamda.2017.11.003, 29269096

[42] Levasseur M Généreux M Bruneau JF Vanasse A Chabot É Beaulac C . Importance of proximity to resources, social support, transportation and neighborhood security for mobility and social participation in older adults: results from a scoping study. BMC Public Health. (2015) 15:503. doi: 10.1186/s12889-015-1824-0, 26002342 PMC4460861

[43] World Health Organization. International Classification of Functioning, Disability and Health (ICF). Geneva: WHO (2001).

[44] Ingold T. Culture on the ground: the world perceived through the feet. J Mater Cult. (2004) 9:315–40. doi: 10.1177/1359183504046896

[45] Solnit R. Wanderlust: A History of Walking. New York: Viking (2000).

[46] Beauchamp MK Hao Q Kuspinar A Amuthavalli Thiyagarajan J Mikton C Diaz T . A unified framework for the measurement of mobility in older persons. Age Ageing. (2023) 52:iv82–5. doi: 10.1093/ageing/afad125, 37902518 PMC10615050

[47] Giles-Corti B Vernez-Moudon A Reis R Turrell G Dannenberg AL Badland H . City planning and population health: a global challenge. Lancet. (2016) 388:2912–24. doi: 10.1016/S0140-6736(16)30066-6, 27671668

[48] Marmot M Friel S Bell R Houweling TAJ Taylor S. Closing the gap in a generation: health equity through action on the social determinants of health. Lancet. (2008) 372:1661–9. doi: 10.1016/S0140-6736(08)61690-6, 18994664

[49] WHO Commission on Social Determinants of Health. Closing the Gap in a Generation: Health Equity Through Action on the Social Determinants of Health. Geneva: World Health Organization (2008). 10.1016/S0140-6736(08)61690-6 18994664

[50] Nieuwenhuijsen MJ. Urban and transport planning pathways to carbon neutral, liveable and healthy cities; a review of the current evidence. Environ Int. (2020) 140:105661. doi: 10.1016/j.envint.2020.105661, 32307209

